# Signet Cell Carcinoma of Colon in a Nineteen-Year-Old Patient: A Case Report

**DOI:** 10.1155/2013/695450

**Published:** 2013-02-19

**Authors:** Özgül Pamukçu, Fatih Selcukbiricik, Ahmet Bilici, Damlanur Sakız, Osman Özdoğan, Fatih Borlu

**Affiliations:** ^1^Department of Internal Medicine, Şişli Etfal Training Hospital, 34360 Istanbul, Turkey; ^2^Department of Medical Oncology, Şişli Etfal Training Hospital, 34360 Istanbul, Turkey; ^3^Department of Pathology, Şişli Etfal Training Hospital, 34360 Istanbul, Turkey; ^4^Department of Gastroenterohepatology, Şişli Etfal Training Hospital, 34360 Istanbul, Turkey

## Abstract

Signet cell carcinoma, which is a subtype of adenocarcinoma, usually originates from the stomach. However, it can also originate from the colon, rectum, gallbladder, pancreas, urinary bladder, and breast. We represent a 19-year-old boy diagnosed with signet cell tumour while he was being evaluated for an initial diagnosis of inflammatory bowel disease.

## 1. Introduction 

Globally, CRC is the third most commonly diagnosed cancer in males and the second in females, with over 1.2 million new cases and 608,700 deaths estimated to have occurred in 2008 [1]. According to the World Health Organization data, about 608.000 deaths from colorectal cancer are estimated worldwide, accounting for 8% of all cancer deaths, making it the fourth most common cause of death from cancer [2].

Colorectal cancer is a common malignancy in adults which peaks around 6th or 7th decades of life. However, less than 20% of the colorectal cancer cases can be seen before the age of 50 [3]. And in USA it was reported that less than 1% of all cancers are colorectal cancers in the first two decades of life [4, 5]. While signet cell carcinoma is a poor differentiated type of adenocarcinoma and behaves more aggresively than ordinary adenocarcinoma of colon, it is more common in younger patients (especially under 40) compared to other types [6].

Because of the lack of awareness at early age and the aggressive characteristic of the tumour, signet ring cell carcinomas of the colon mostly present as advanced stage. In this case report, we report a 19-year-old boy who we were evaluating for an initial diagnosis of inflammatory bowel disease was our first diagnosis. We have interned patient with that initial diagnosis.

## 2. Case

**Figure 1:**
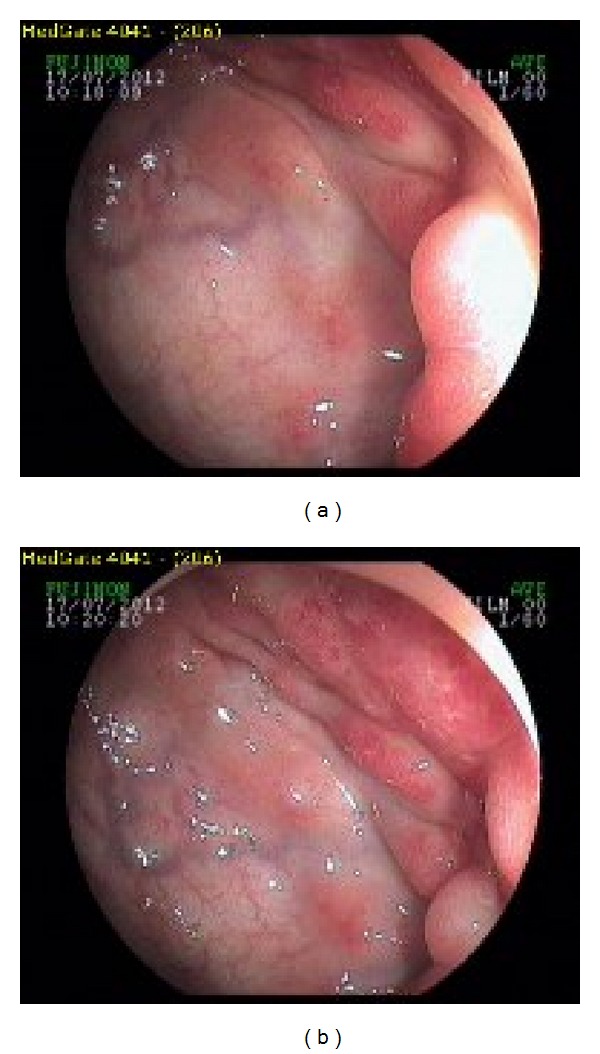
(a), (b) Colonoscopic view of the signet cell tumour in sigmoid colon. The colonoscopy could not been completed according to the obstruction.

**Figure 2:**
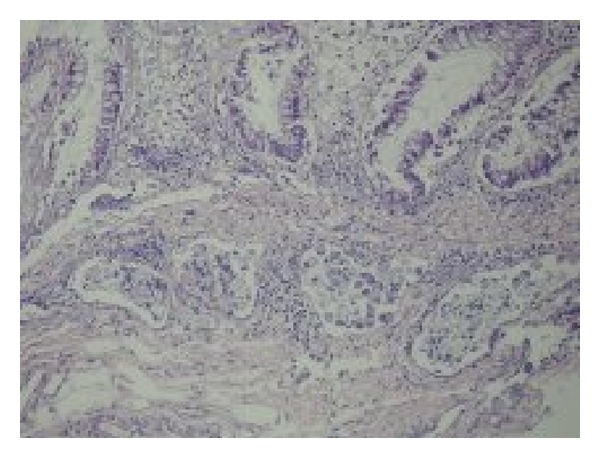
A number of signet cells regarding signet cell carcinoma can be seen in the submucosal layer of vascular structures. (H&E; 100x).

A 21-year-old boy who has been evaluated in emergency service with abdominal pain was referred to our outpatient clinic because of the reason that there was seen edema and inflammation around the ileal wall and some collection regarding the initial diagnosis of inflammatory bowel disease in the CT scan performed to exclude acute abdomen. The patient has no family history of any cancer. In his physical examination, mildtenderness has been detected in the right lower quadrant and periumblical site of the abdomen. His bowel sounds were normal and in his rectal examination, there was a formed stool without blood. In his laboratory results, pathological results were as follows: CRP: 25; ESR: 22 and fecal occult blood test was positive. In his CT/CT enterography there was seen free subhepatic and pelvic abdominal fluid; asimetric thickening in the sigmoid colon walls; derangement of bowels and peritoneum. According to these findings the radiologists pointed that it must be evaluated not only for inflammatory bowel disease but also for the diseases involve peritoneum. Then colonoscopy and gastroscopy were performed and while there was no pathological sign in gastroscopy, an obstruction in sigmoid colon has been detected by colonoscopy (Figures 1(a), and 1(b)). The biopsies were taken. Before the pathological investigation has been completed, patient was presented with subileus according to the obstruction of sigmoid colon. Placement of a metal stent to the sigmoid colon was tried but it was not successful according to the hardness of the tumour. Biopsy results were reported as signet ring cell carcinoma of sigmoid colon (Figure 2). Signet cell carcinoma cells were also seen in the abdominal fluid which we have obtained after the pathological diagnosis. Afterwards, the PET/CT scan was performed and it also showed peritonitis carcinomatosa with a significant omental cake. As the patient was accepted as inoperable with an advanced stage, patient underwent the surgery for the palliative colostomy. FOLFOX regimen which includes 5 Fluoruracil, calcium folinate, oxaliplatin, and bevacizumab (FOLFOX + Bevacizumab) has been started with the diagnosis of metastatic colon cancer. The patient is still undergoing treatment.

## 3. Discussion

Signet cell cancer of the colon is a rare subtype of colon cancer, where abundant intracytoplasmic mucin pushes the nucleus to the periphery giving a signet ring appearance. Primary colorectal signet cell carcinoma is diagnosed when the following criteria are satisfied. Firstly, the tumor must be primary, histological material must be adequate and signet ring cells present in more than 50% of the cancer [7]. Our patient could have been accepted as a primary signet cell carcinoma according to these criteria as we have ruled out any cancer in another primary site.

More than 96% of signet-ring cell carcinomas arise in the stomach, and the rest occurred in other organs [8]. Signet ring cell carcinoma accounts for less than 1% of all colon cancers [9]. 

Primary signet-ring cell carcinoma of the colon and rectum which was described by Laufman and Saphir in 1951 [11] is so rare that its incidence was reported as 0.01%–2.6% [12]. 

Signet ring cell tumours have an aggressive clinical course and a poor prognosis. There is high incidence of peritoneal metastases and relatively low incidence of hepatic metastases, a characteristic feature distinguishing colorectal signet-ring cell carcinoma from nonsignet colorectal carcinoma [13].

In the literature, signet-ring cell carcinomas tend to affect predominately young individuals and HNPCC patients [9]. However Zambrano et al. reported that they have not found relation between the HNPCC and primary signet cell carcinoma of rectum and colon [14]. Patients are often noted to be younger compared to the patients with non-signet cell tumors of the colon. Median age is about 59 years when compared to the nonsignet cell cancer, where the median age is about 61 years [15]. Our patient was one of the youngest patients in literature excluding the the pediatric population where 68% of all colorectal cancers show mucinous characteristics reported by the review of Hill et al. [16]. However also in children a limited number of colorectal carcinoma have been reported and while most of these cases are teens, the youngest one reported was a 9-month-old baby [17]. The cases with colon cancer in young patients in the literature are shown in the Table 1.

The most common presenting symptom in colorectal signet cell tumour is abdominal pain. Other symptoms include rectal bleeding, change on bowel habits, and weight loss [18]. The symptoms can mimic inflammatory bowel disease. Our patient's symptoms were not only similar to inflammatory bowel disease clinically with the rectal bleeding and abdominal pain; but also radiologically the initial CT scan let us considering inflammatory bowel disease. 

In 2005, Achneck et al. also reported a signet ring cell carcinoma case which was treated as an inflammatory bowel disease initially [19]. The infrequency of the disease among the young population makes the diagnosis more difficult and the prognosis less favorable.

As it was reported that the intestinal type—especially the signet cell type—of gastric cancers has increased over the last 50 years, it must be considered in the patients present with persistent abdominal pain even if the patient is young [15].

## Figures and Tables

**Table 1:** Reported cases with colon cancer in young patients.

	Age-sex (m: male f: female)	Symptom	Site of tumour and histology	Treatment	Survival
Shih et al. [2]	15, m	Epigastric pain, decreased apetite, mild watery diarrhea	Hepatic flexura of the colon/mucinous type adenocarcinoma	Palliative ileosigmoidostomy Chemo-ND*	1 month after surgery
Tung et al. [6]	31 y, m	Persistent abdominal pain, vomiting Subacute intestinal obstruction	Rectosigmoid side of colon signet ring cell carcinoma	Radical surgical resection Chemo-ND	ND*
Messerini et al. [7]	10 y, f	Subacute intestinal obstruction, recurrent vague abdominal pain	Descending and sigmoid colon/poorly differentiating mucin secreting adenocarcinoma	Left hemicolectomy; 5 FU + Leucovorin	8 months-recurrence free as known. No further information.
Fu et al. [8]	16 y, f	Abdominal pain, rectal bleeding	Sigmoid colon/signet ring cell carcinoma	Hemicolectomy + anterior resection Chemo-ND	ND
Thota et al. [9]	17 y, m	Progressive right sided abdominal pain and swelling	Ascending colon/signet ring cell carcinoma	Right colon resection + ileocolic anastomosis FOLFOX-6	1 year
Ko et al. [10]	13, m	Abdominal pain, poor apetite, abdominal fullness	Ascending colon/signet cell carcinoma	Right hemicolectomy; 5 FU + Leucovorin Levamisole Sisplatin	1 year, 5 months.
Özgül et al.	19 y, m	Persistant abdominal pain, weight loss	Sigmoid colon/signet ring cell carcinoma	Palliative colostomy FOLFOX-6 + bevacizumab	ND

*ND: not determined.
